# Supplementary material for: Mold Fungal Resistance of Loose-Fill Thermal Insulation Materials Based on Processed Wheat Straw, Corn Stalk and Reed
Source: Polymers (Basel). 2024 Feb 19;16(4):562. doi: 10.3390/polym16040562 (PMC10892266; doi:10.3390/polym16040562)
Supplement: Supplementary file 1 [file polymers-16-00562-s001.zip › polymers-2853738-supplementary.pdf]

polymers

MDPI

Supplementary Materials

Mold Fungal Resistance of Loose-Fill Thermal Insulation Materials Based on Processed Wheat Straw, Corn Stalk and Reed

Ramunas Tupciauskas [1,*], Zigmunds Orlovskis [2,3], Karlis Trevors Blums [2,3], Janis Liepins [2], Andris Berzins [1,4], Gunars Pavlovics [1] and Martins Andzs [1]

1 Laboratory of Biorefinery, Latvian State Institute of Wood Chemistry, Dzerbenes 27, 1006 Riga, Latvia; andris.berzins@kki.lv (A.B.); pavlovichs@inbox.lv (G.P.); martins.andzs@kki.lv (M.A.)
2 Faculty of Biology, University of Latvia, Jelgavas 1, 1004 Riga, Latvia; zigmunds.orlovskis@lu.lv (Z.O.); ktblums@gmail.com (K.T.B.); janis.liepins@lu.lv (J.L.)
3 Latvian Biomedical Research and Study Centre, Ratsupites 1 k-1, 1067 Riga, Latvia
4 Faculty of Forest and Environmental Sciences, Latvia University of Life Sciences and Technologies, Akademijas 11, 3001 Jelgava, Latvia
* Correspondence: ramunas.tupciauskas@kki.lv

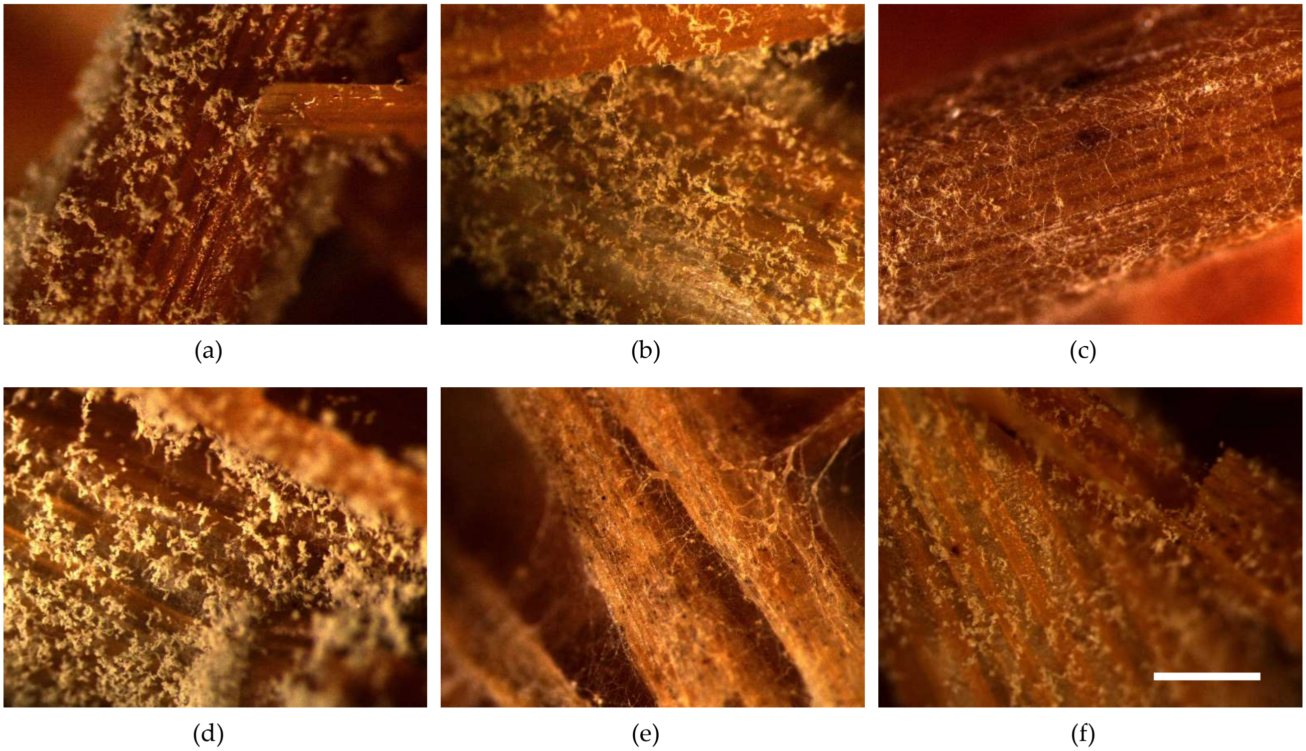

Figure S1. Microscopical surface view (25×, scale bar 1 mm) of WS-raw samples after 4 weeks incubation colonized with: (**a**) *T. viride*, (**b**) *C. globulosum*, (**c**) *P. variotii*, (**d**) *P. pinophilum*, (**e**) *A. niger*, (**f**) control (H2O).

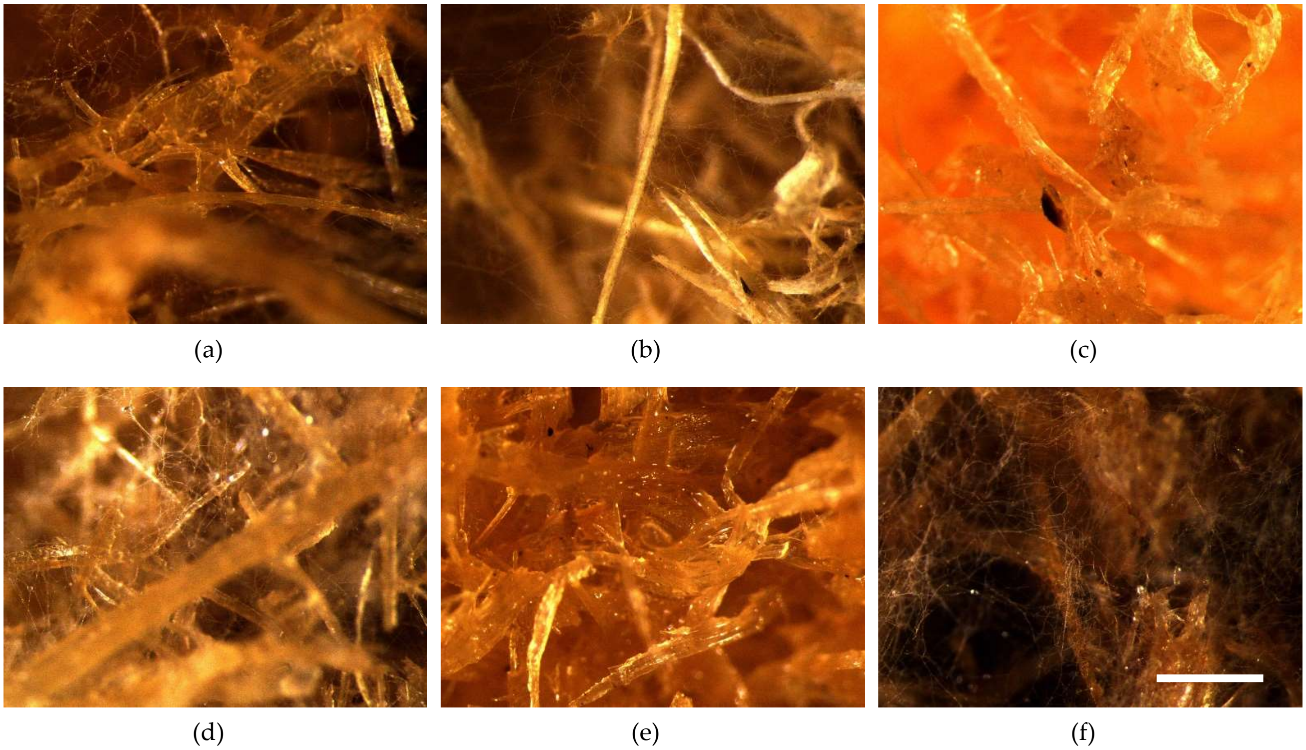

Figure S2. Microscopical surface view (25×, scale bar 1 mm) of WS-TMP samples after 4 weeks incubation colonized with: (**a**) *T. viride*, (**b**) *C. globulosum*, (**c**) *P. variotii*, (**d**) *P. pinophilum*, (**e**) *A. niger*, (**f**) control (H2O).

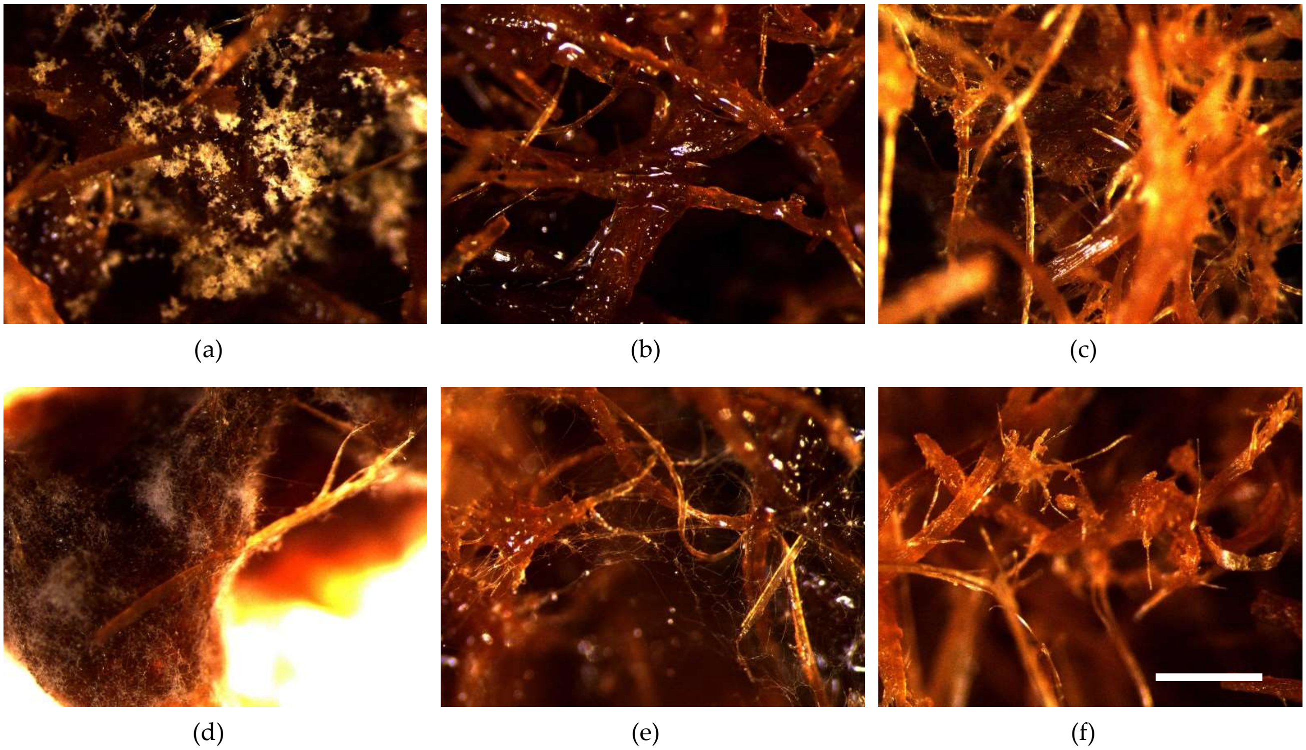

Figure S3. Microscopical surface view (25×, scale bar 1 mm) of WS-SE samples after 4 weeks incubation colonized with: (**a**) *T. viride*, (**b**) *C. globulosum*, (**c**) *P. variotii*, (**d**) *P. pinophilum*, (**e**) *A. niger*, (**f**) control (H2O).

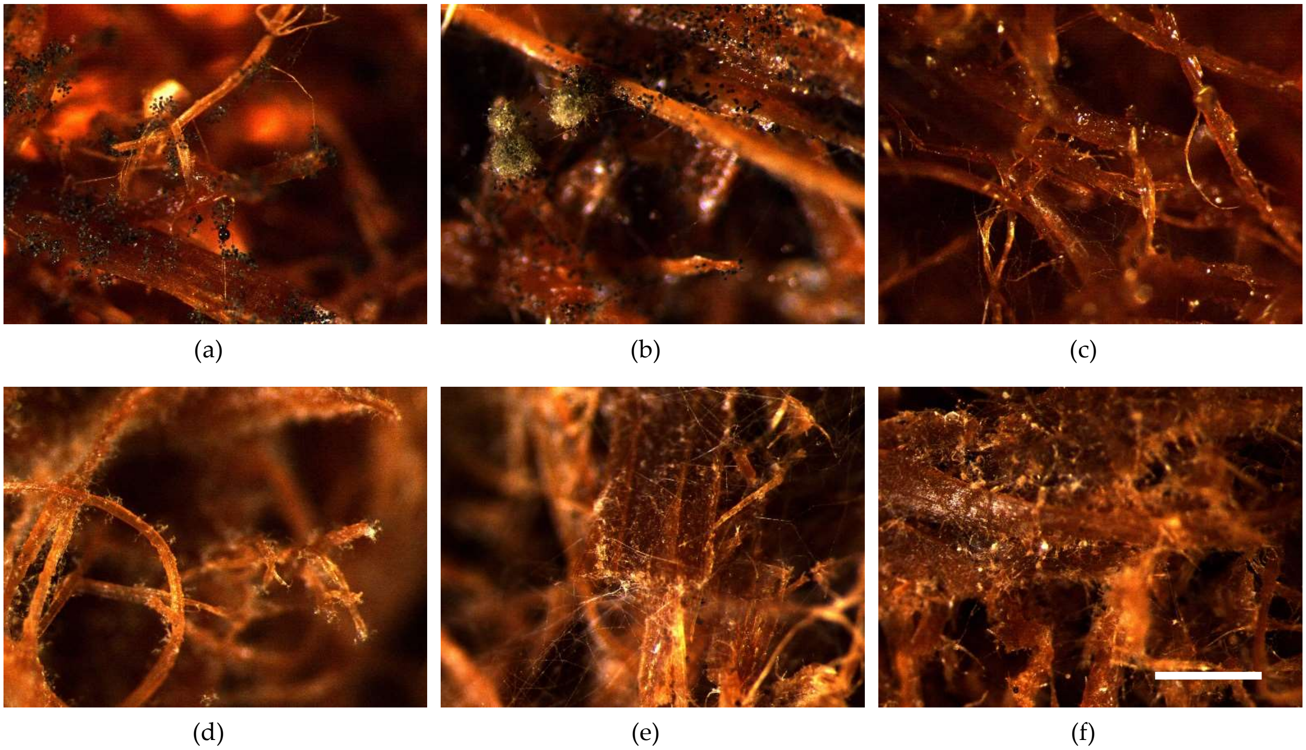

Figure S4. Microscopical surface view (25×, scale bar 1 mm) of WS-SE* samples after 4 weeks incubation colonized with: (**a**) *T. viride*, (**b**) *C. globulosum*, (**c**) *P. variotii*, (**d**) *P. pinophilum*, (**e**) *A. niger*, (**f**) control (H2O).

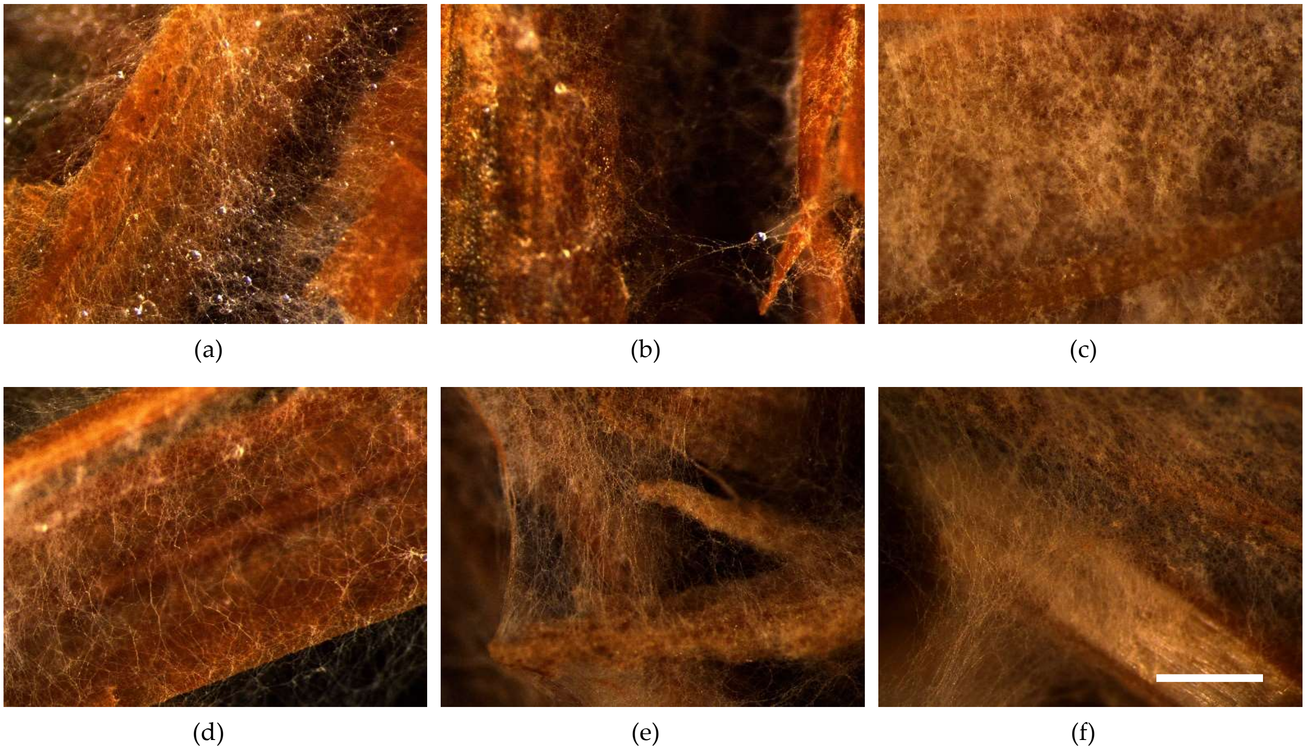

Figure S5. Microscopical surface view (25×, scale bar 1 mm) of Corn-raw samples after 4 weeks incubation colonized with: (**a**) *T. viride*, (**b**) *C. globulosum*, (**c**) *P. variotii*, (**d**) *P. pinophilum*, (**e**) *A. niger*, (**f**) control (H2O).

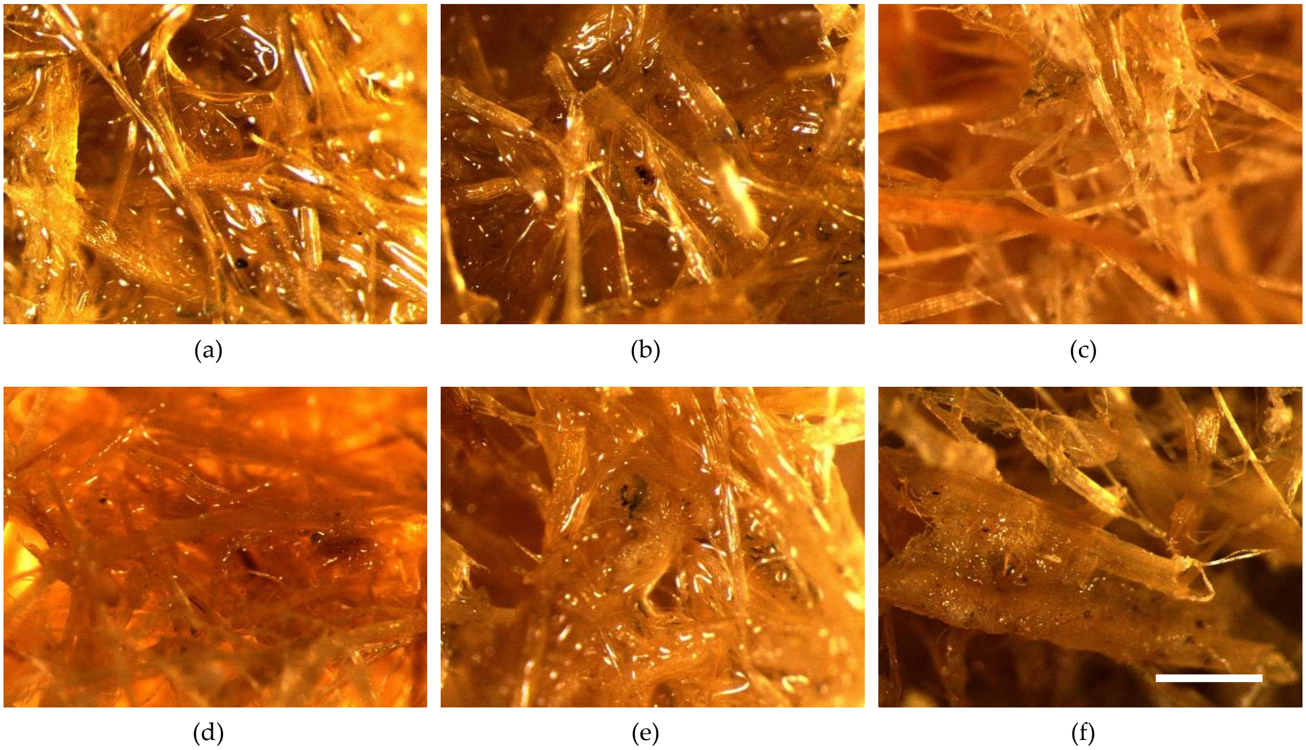

Figure S6. Microscopical surface view (25×, scale bar 1 mm) of Corn-TMP samples after 4 weeks incubation colonized with: (**a**) *T. viride*, (**b**) *C. globulosum*, (**c**) *P. variotii*, (**d**) *P. pinophilum*, (**e**) *A. niger*, (**f**) control (H2O).

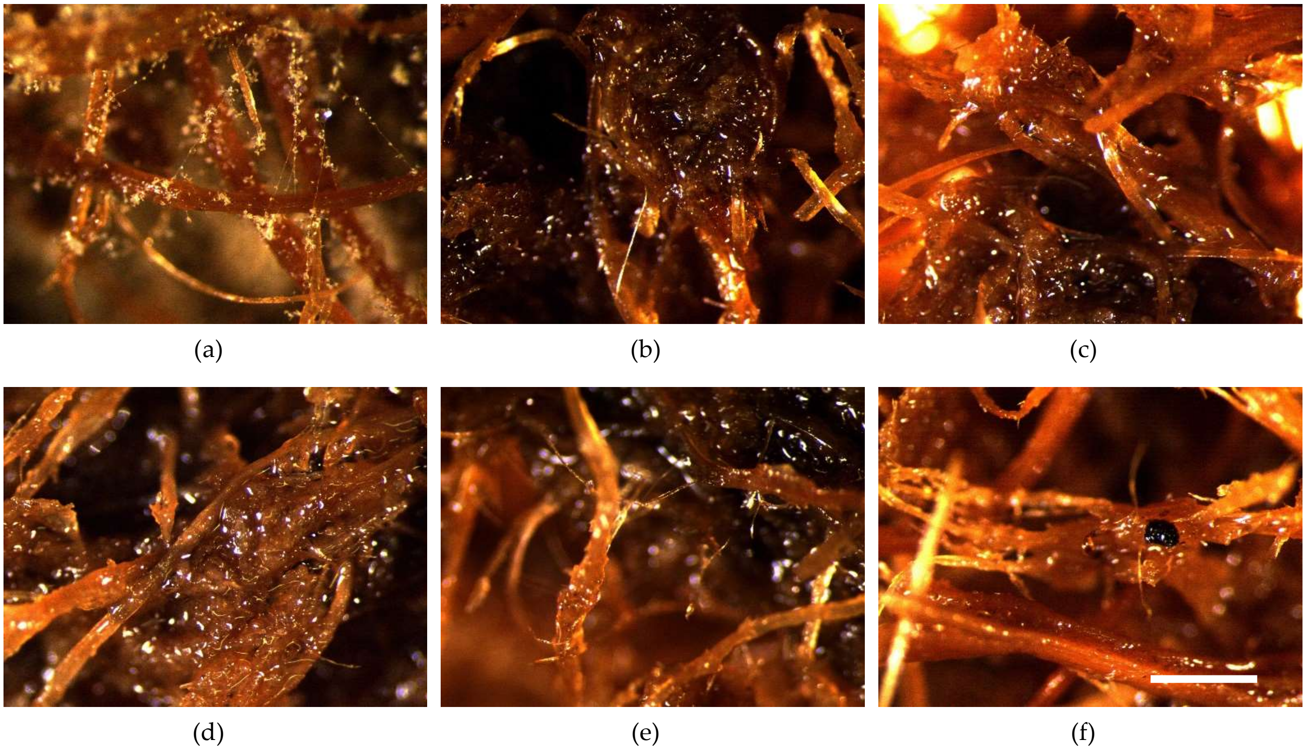

Figure S7. Microscopical surface view (25×, scale bar 1 mm) of Corn-SE samples after 4 weeks incubation colonized with: (**a**) *T. viride*, (**b**) *C. globulosum*, (**c**) *P. variotii*, (**d**) *P. pinophilum*, (**e**) *A. niger*, (**f**) control (H2O).

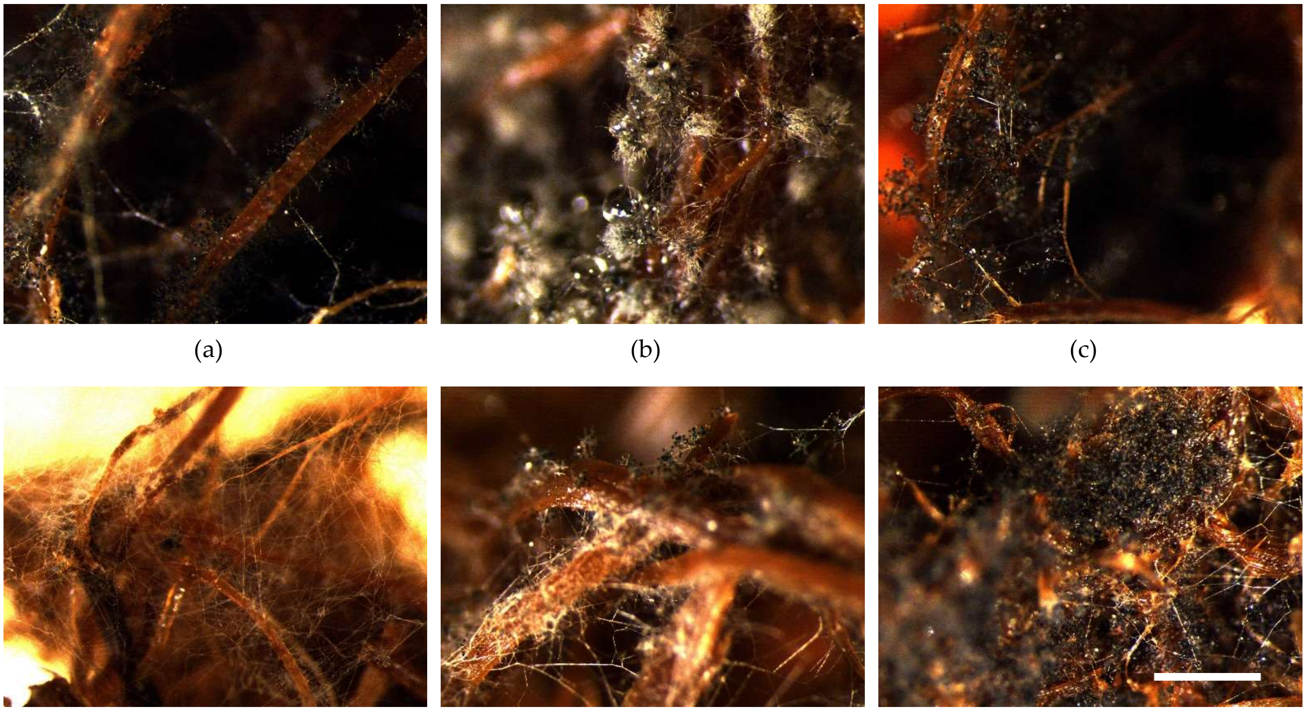

(a) (b) (c)

(d) (e) (f)

Figure S8. Microscopical surface view (25×, scale bar 1 mm) of Corn-SE* samples after 4 weeks incubation colonized with: (**a**) *T. viride*, (**b**) *C. globulosum*, (**c**) *P. variotii*, (**d**) *P. pinophilum*, (**e**) *A. niger*, (**f**) control (H2O).

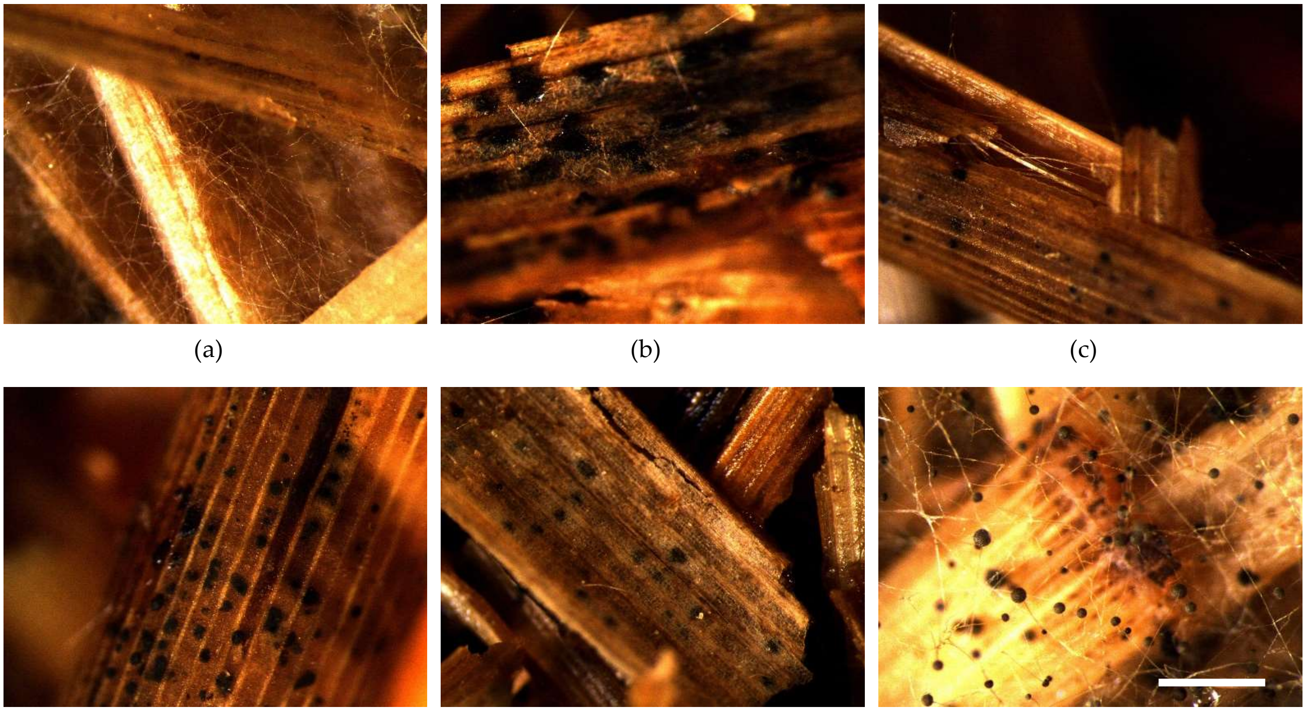

(a) (b) (c)

(d) (e) (f)

Figure S9. Microscopical surface view (25×, scale bar 1 mm) of Reed-raw samples after 4 weeks incubation colonized with: (**a**) *T. viride*, (**b**) *C. globulosum*, (**c**) *P. variotii*, (**d**) *P. pinophilum*, (**e**) *A. niger*, (**f**) control (H2O).

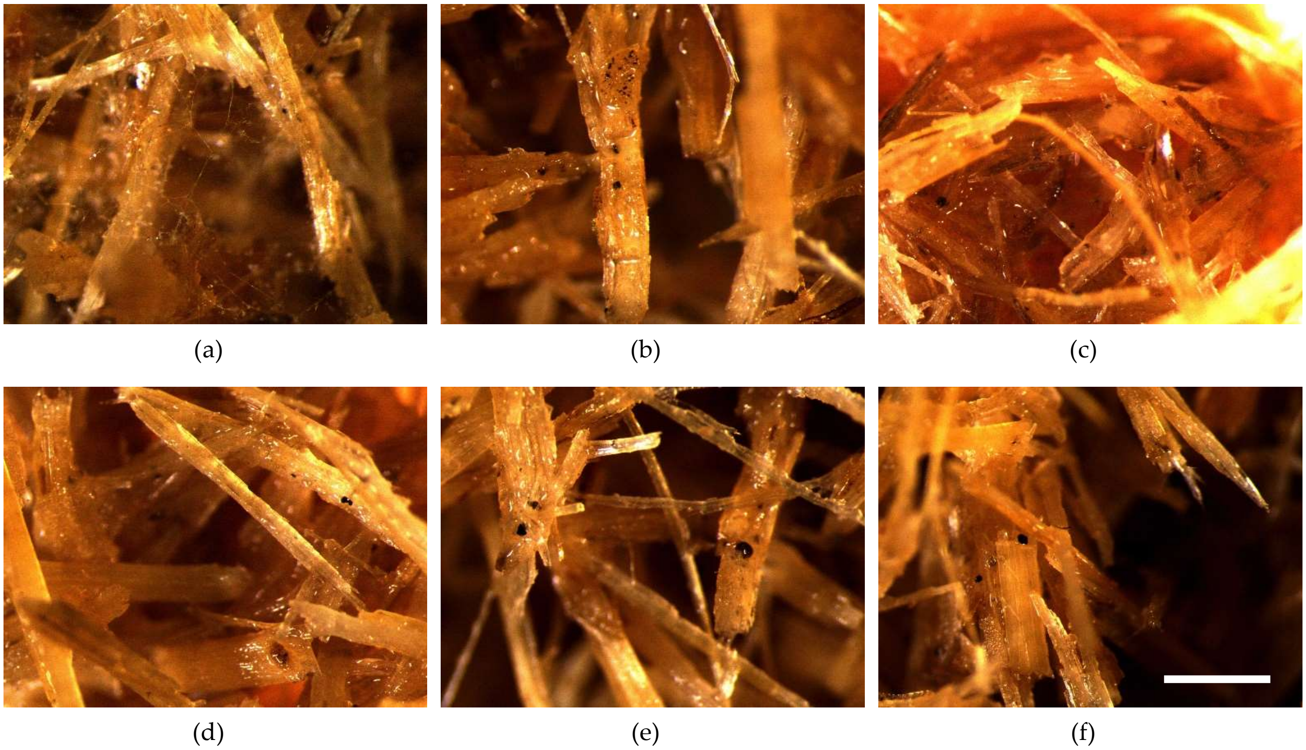

Figure S10. Microscopical surface view (25×, scale bar 1 mm) of Reed-TMP samples after 4 weeks incubation colonized with: (**a**) *T. viride*, (**b**) *C. globulosum*, (**c**) *P. variotii*, (**d**) *P. pinophilum*, (**e**) *A. niger*, (**f**) control (H2O).

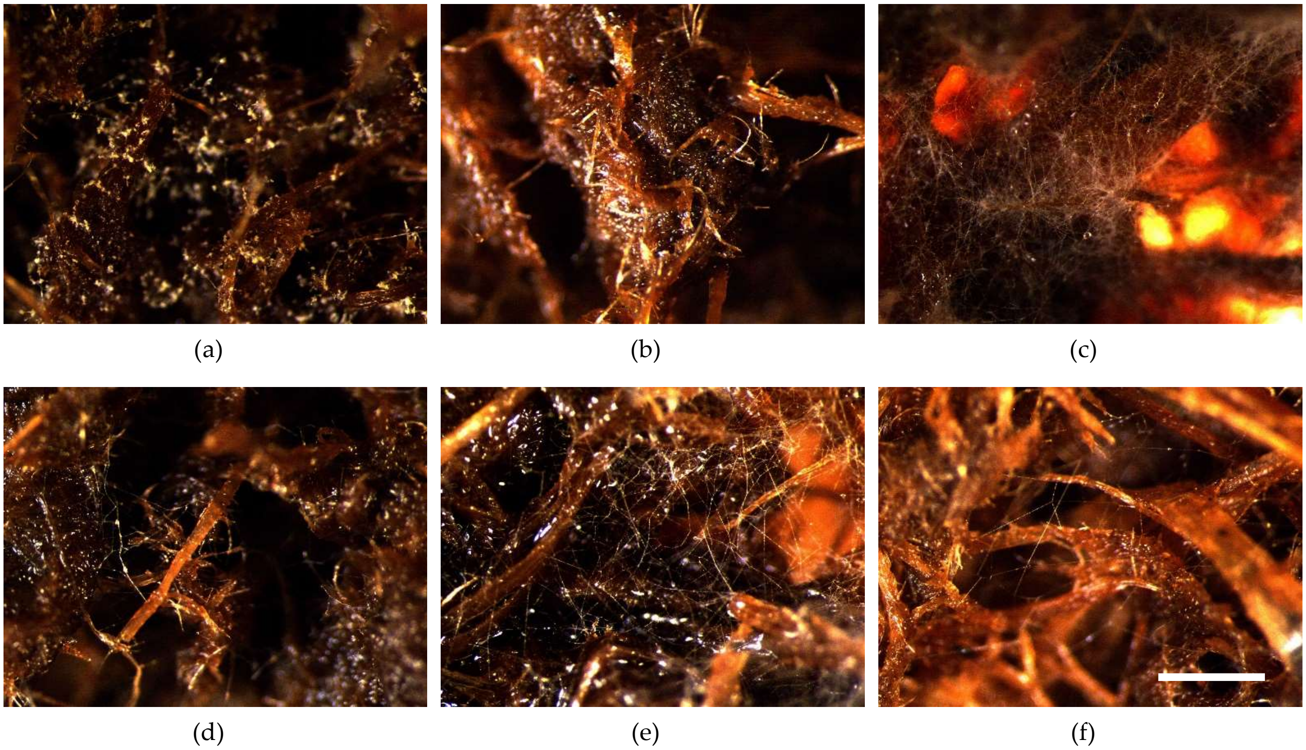

Figure S11. Microscopical surface view (25×, scale bar 1 mm) of Reed-SE samples after 4 weeks incubation colonized with: (**a**) *T. viride*, (**b**) *C. globulosum*, (**c**) *P. variotii*, (**d**) *P. pinophilum*, (**e**) *A. niger*, (**f**) control (H2O).

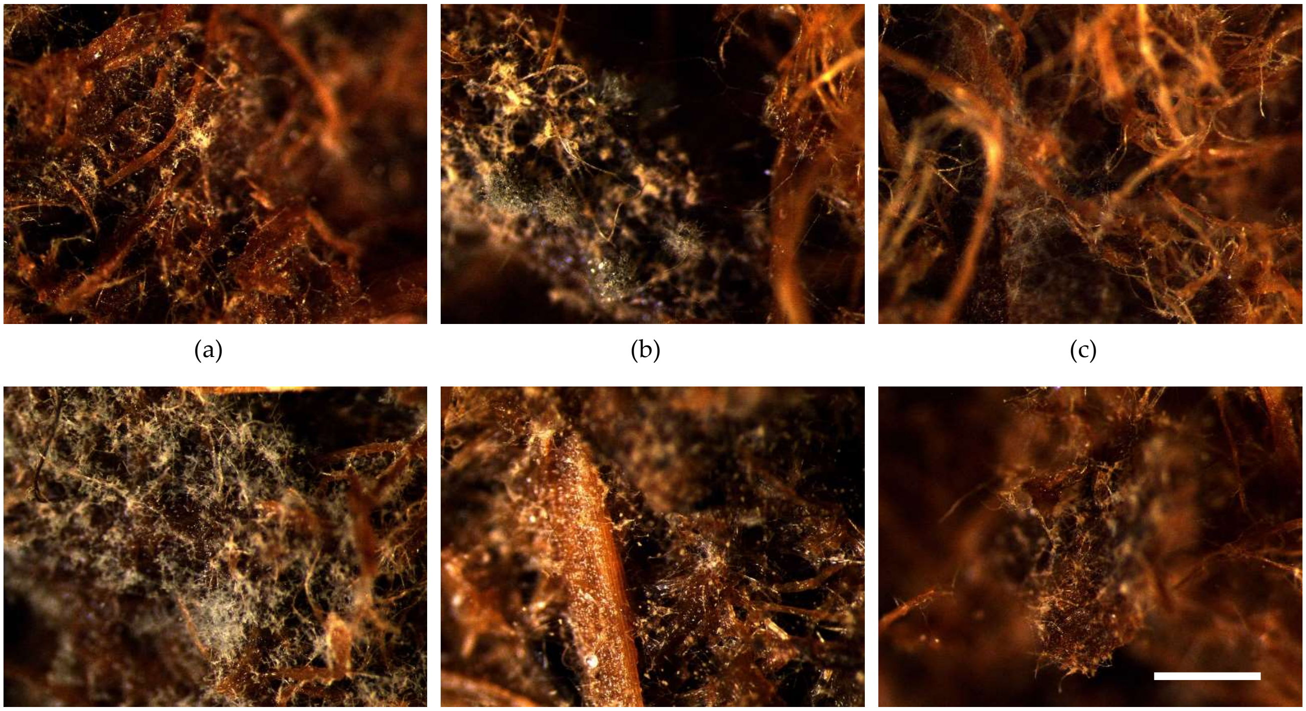

(a) (b) (c)

(d) (e) (f)

Figure S12. Microscopical surface view (25×, scale bar 1 mm) of Reed-SE* samples after 4 weeks incubation colonized with: (**a**) *T. viride*, (**b**) *C. globulosum*, (**c**) *P. variotii*, (**d**) *P. pinophilum*, (**e**) *A. niger*, (**f**) control (H2O).
